# Origin and Quenching of Novel ultraviolet and blue emission in NdGaO3: Concept of Super-Hydrogenic Dopants

**DOI:** 10.1038/srep36352

**Published:** 2016-11-03

**Authors:** Siddhartha Ghosh, Surajit Saha, Zhiqi Liu, M. Motapothula, Abhijeet Patra, Nikolai Yakovlev, Yao Cai, Saurav Prakash, Xiao Hu Huang, Chuan Beng Tay, Chun Xiao Cong, Thirumaleshwara Bhatt, Surani B. Dolmanan, Jianqiang Chen, Weiming Lü, Zhen Huang, Sudhiranjan Tripathy, Soo Jin Chua, Ting Yu, Mark Asta, A. Ariando, T. Venkatesan

**Affiliations:** 1 NUSNNI-Nanocore, National University of Singapore, 117411, Singapore; 2 Department of Physics, National University of Singapore, 117542, Singapore; 3 School of Materials Science and Engineering, Beihang University, Beijing, 100191, China; 4 Institute of Materials Research and Engineering, A*STAR (Agency for Science, Technology, and Research), Innovis 08-03, 2 Fusionopolisway, 138634, Singapore; 5 Department of Materials Science and Engineering, UC Berkeley, 210 Hearst Ave, Berkeley, CA, 94720, United States; 6 Department of Electrical and Computer Engineering, National University of Singapore, 117576, Singapore; 7 Divsion of Physics and Applied Physics, School of Physical and Mathematical Sciences, Nanyang Technological University, 637371, Singapore

## Abstract

In this study we report the existence of novel ultraviolet (UV) and blue emission in rare-earth based perovskite NdGaO_3_ (NGO) and the systematic quench of the NGO photoluminescence (PL) by Ce doping. Study of room temperature PL was performed in both single-crystal and polycrystalline NGO (substrates and pellets) respectively. Several NGO pellets were prepared with varying Ce concentration and their room temperature PL was studied using 325 nm laser. It was found that the PL intensity shows a systematic quench with increasing Ce concentration. XPS measurements indicated that nearly 50% of Ce atoms are in the 4+ state. The PL quench was attributed to the novel concept of super hydrogenic dopant (SHD)”, where each Ce^4+^ ion contributes an electron which forms a super hydrogenic atom with an enhanced Bohr radius, due to the large dielectric constant of the host. Based on the critical Ce concentration for complete quenching this SHD radius was estimated to be within a range of 0.85 nm and 1.15 nm whereas the predicted theoretical value of SHD radius for NdGaO3 is ~1.01 nm.

Doping is a process in which atomic impurities are intentionally added to a host material to modify its properties. It has had a revolutionary impact in changing or introducing electronic^1^,^2^, magnetic^3^,^4^, optical^5^,^6^, and catalytic^7^ properties for various applications. Transition Metal Oxides with strongly correlated electron systems usually have complex electronic phase diagrams that are exceptionally sensitive to the orbital occupancy of electrons and chemical/carrier doping. Electronic phase transitions observed in ABO_3_ perovskite systems and their interfaces^8^,^9^ are of broad interest in materials physics to understand both fundamentally appealing band structure and exciting potential applications in oxide electronic devices^10^. Specifically, the monotonic shift of carrier transport and magnetic properties with various 4*f* rare earth lanthanide elements correlated with the steric effect found in the rare-earth based perovskites like *R*BO_3_ (where *R* = rare earth lanthanide elements) can act as an excellent platform for revealing the underlying strong correlation physics^11^.

In this study we report a novel electronic doping strategy via super-hydrogenic dopants in oxides which can be a very interesting approach for fundamental understanding as well as potential applications. As we know, the radius of hydrogen atom in free space is about 0.5 Å; but the whole situation changes when this atom is placed into a high dielectric constant material. The Rydberg radius of that atom becomes much larger - the free space radius multiplied by the dielectric constant of the system, these kind of super-hydrogenic atoms can be realized in wide band-gap oxides (with high dielectric constants) by doping them with *n* or *p* type dopants. It has been also predicted that at very low concentrations (of about only 1%) these super hydrogenic orbitals will overlap with each other to create a band and this band may cause magnetic (ferro or antiferro) effects in the material^12^,^13^. Along with magnetic effects, novel transport and optical properties can also be found in these systems. In addition to our current study involving NdGaO_3_ (NGO), this novel approach can be also utilized to study other exotic materials like rare-earth oxides (mostly with R_2_O_3_ formula) which exhibit large band-gap, high dielectric constant with potential applications in electronic and photonic technologies. In this paper we report the origin of room-temperature ultraviolet (UV) and blue emission in NdGaO_3_ (NGO) bulk systems and the subsequent quench of PL by Ce^4+^ doping in the place of Nd^3+^ ions in NGO as a case study of super-hydrogenic dopant (SHD).

NGO is a paramagnetic insulator, which undergoes a magnetic phase transition into an antiferromagnetic phase at ~1 K due to strong super-exchange interaction^14^. It has an orthorhombic perovskite structure with lattice constants *a* = 5.43 Å, *b* = 5.50 Å and *c* = 7.71 Å while its orthorhombically distorted pseudo cubic cell has a lattice constant of 3.87 Å. It is close to lattice constants of many perovskite materials and therefore generally used as substrates for colossal magneto-resistance thin film and other perovskite thin film growth. Due to the very low dielectric loss of NGO in the GHz frequency range^15^, it has been extensively utilized as substrates for high-temperature superconducting film fabrications in microwave devices as well.

Rare earth based oxides, perovskites and their interfaces are of great interest in optical studies as rare earth ions can serve as emission centers for laser systems, such as Nd:YAG (Nd-doped Y_3_Al_5_O_12_)^16^,^17^. The photoluminescence (PL) properties of the Nd^3+^ ion in NGO single crystals were studied by Orera *et al.*^18^, where the samples were excited by light from a tungsten lamp. But, no emission was detected in NGO even at low temperatures. In this letter, we report for the first time ultraviolet (UV) and blue emission in NGO single crystals and poly-crystalline bulk samples obtained by the excitation of a 325 nm laser. There are two bands of UV emission peaks around 360 and 390 nm while blue emission peaks are centered at 420 nm. Further, we have seen a drop in the PL intensity of the sharp blue emission lines at 390 nm by systematic doping of Ce in NGO poly-crystalline bulk samples. This PL quench can be explained by the concept of “super-hydrogenic dopant”.

## Results

### Material Characterization

**Figure 1:**
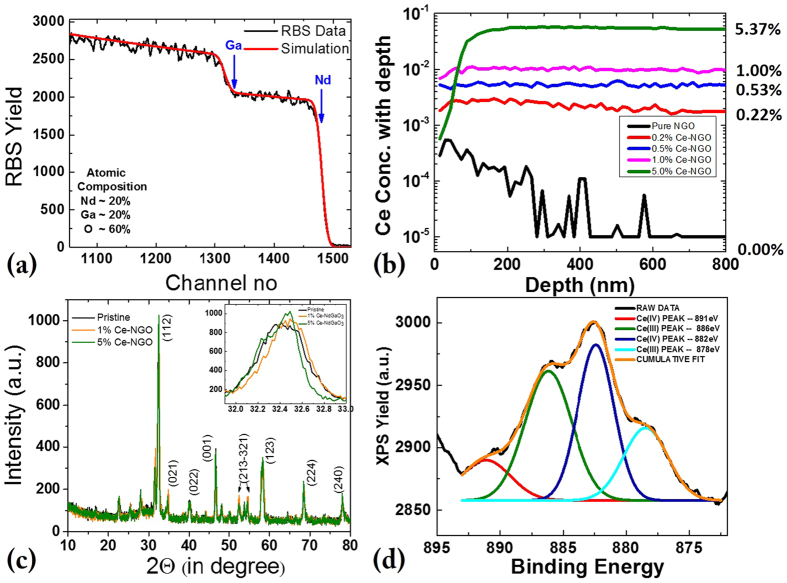
Structural and Compositional characterization of poly-crystalline bulk NGO samples (**a**) RBS analysis of pure NGO shows sharp Ga and Nd edges, (**b**) SIMS study showing Ce doping percentages, (**c**) powder XRD measurement showing NdGaO3 peaks, (**d**) XPS analysis showing presence of both Ce(III) and Ce(IV) states in Ce doped NGO bulk samples.

Figure 1 shows elemental composition and structural analysis of an NGO poly-crystalline bulk sample. We have used Rutherford Backscattering Spectroscopy (RBS) to calculate the composition of NdGaO_3_ target and Fig. 1(a) shows a best simulation fit which clarifies that for pure NdGaO_3_ bulk samples the elemental composition was ideal – Nd:Ga:O ≈1:1:3. Further RBS and Particle Induced X-ray Emission Spectroscopy (PIXE) were used to find the Ce dopant level in doped NGO poly-crystalline samples. Unfortunately, Ce elemental level measurements using RBS and PIXE were not possible as the atomic mass or Ce (Z = 57) and Nd (Z = 58) are comparable and thus the signals of Ce and Nd overlap with each other in both RBS and PIXE spectra. Followed by RBS-PIXE, we instead tried the Secondary Ion Mass Spectroscopy (SIMS) technique for quantifying the Cerium dopant in the doped NGO samples [Fig. 1(b)]. Five samples were measured, with nominal concentrations of 0%; 0.2%; 0.5%; 1.0%; 5.0%, respectively. Numbers in the Fig. 1(b) mean that 0.2% Ce doped NGO has ~0.22% Ce doping & 0.5% Ce doped NGO target has ~0.53% Ce doping and so on. The measurements show that the actual concentration of Ce correlates very well with the desired values in general. Initially it was found that the SIMS peak of Ce (140) overlaps with peak of Ga2 (69 + 71), so all the final values were obtained after decomposing of Ce (140) and Ga2 (69 + 71) overlapping peaks. It is also important to note that in most of the doped bulk samples (except in the 5% doped sample) the Ce concentration is very uniform throughout the samples up to a thickness level of 800 nm, while in the 5% Ce-NGO sample the Ce concentration is low on the surface but then from about 100 nm thickness the Ce concentration level reaches the desired 5% and remains uniform over the next 700 nm. In Fig. 1(c) we have shown the structural analysis of pure and doped NGO samples using X-ray Diffraction technique (XRD). All doped samples have been studied for structural information, here we have plotted powder XRD patterns of the pure, the most highly doped (5%) and one intermediate (1%) doped NGO bulk samples. In theta-2theta scans from 20 to 80° we find various different XRD peaks of NGO, among which the (112), (001), (123) and (024) peaks are prominent. Under magnified XRD scans for the (112) peak of [Fig. 1(c)-inset], it can be seen that the peak shifts slightly towards a higher angle with Ce doping, which indicates a smaller lattice spacing and can be attributed to the smaller ionic radii of Ce^4+^ ion (0.97 Å or 1.07 Å depending on coordination number 8 or 10) compared to ionic radii of Nd^3+^ in NGO as the co-ordination number of Nd^3+^ in NGO is 9 and the corresponding ionic radius is 1.16 Å, a value ~10% larger. Further analysis was done to realize the electronic state of dopant Ce in the NGO crystal structure, as it is well known that Ce(III) state yields XPS peaks near 880 eV & 886 eV while the Ce(IV) state exhibits XPS peaks near 882 eV & 890 eV. In the actual XPS measurements we found a mixture of all these peaks [see Fig 1(d)], indicating a mixture of Ce^3+^ and Ce^4+^ states (be discussed in detail later)].

### Photoluminescence Study

**Figure 2:**
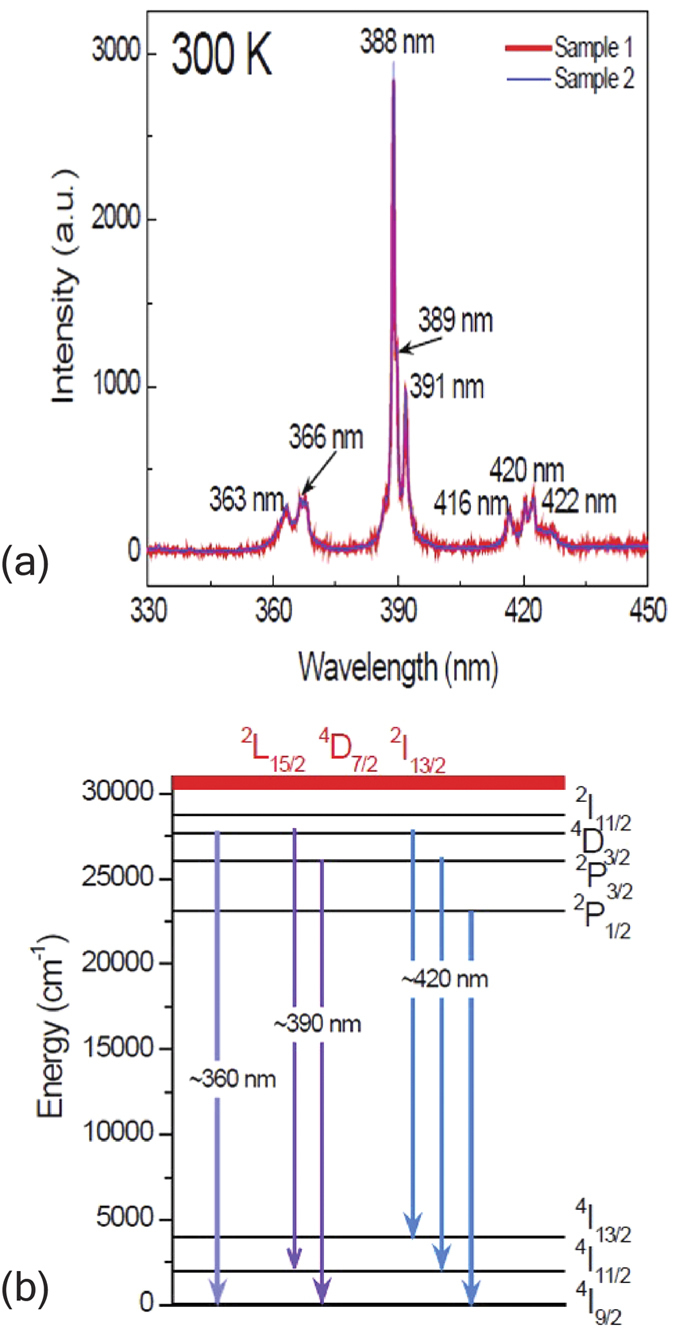
Room-temperature PL emission of NGO single crystals. (**a**) Room-temperature PL spectra of two NdGaO_3_ (NGO) single crystals excited by a 325 nm laser. (**b**) Schematic of possible photoemission in NGO. The red energy levels are overlapping high energy levels.

Room temperature PL spectra were measured in the range of 330 nm to 700 nm. However, no PL was detected above 450 nm, which is consistent with the results reported^18^. Figure 2(a) shows room-temperature PL spectra of two NGO single crystals excited by a laser intensity of 1.5 MW/cm^2^. Generally, PL spectra of NGO single crystals exhibit a very sharp UV emission peak at 388 nm. There are a few of emission peaks, which can overall be categorized into three bands. The first band around 360 and the second one around 390 nm are UV emission peaks while the third one around 420 nm pertains to blue emission. The characteristic energy level diagram of rare earth ions Ln^3+^, known as Dieke diagram only changes slightly in different crystal structures^19^. The wavelength values of three emission centers (360, 390 and 420 nm) correspond to the wavenumbers of 27700, 25600 and 23800 cm^−1^, respectively. The ground state of Nd^3+^ is ^4^I_9/2_. The 325 nm laser excites electrons in the ground state ^4^I_9/2_ to energy levels of ~30769 cm^−1^, which are ^2^L_15/2_, ^4^D_7/2_ and ^2^I_13/2_ levels [schematized in red in Fig. 2(b)]. Then the excited electrons relax into lower energy levels such as ^4^D_3/2_, ^2^P_3/2_ and ^2^P_1/2_. Based on the energy level diagram of Nd^3+^ 17, the PL emission around 360 nm pertains to the optical transition ^4^D_3/2_ → ^4^I_9/2_; the emission around 390 nm corresponds to the optical transitions ^4^D_3/2_ → ^4^I_11/2_ and ^2^P_3/2_ → ^4^I_9/2_; and the emission around 420 nm can be ascribed to the optical transitions ^4^D_3/2_ → ^4^I_13/2_, ^2^P_3/2_ → ^4^I_11/2_ and ^2^P_1/2_ → ^4^I_9/2_. Such possible emission processes are schematized in Fig. 2(b). Invariably, the crystal field in NGO participates in optical emissions, mainly resulting in the energy level and emission peak splitting, which is not included in the schematic. Further systematic studies of room temperature UV-VIS absorption, pump-probe spectroscopy, laser intensity variation and low-temperature PL emission were carried out on NGO single crystals for a better understanding of optical properties of this material (see the Supplementary Material).

It is worthwhile to point out that no photoemission was seen in NGO under the tungsten lamp excitation^18^. Our PL measurements with various laser excitation intensity show that the PL signal is linear with laser excitation intensity (Supplementary Material) and we have used a 325 nm laser with a spot size of ~800 nm and a power of 10 mW, corresponding to an excitation intensity of ~1.5 MW/cm^2^, which could be several orders of magnitude higher than the intensity of the tungsten lamp through a 0.5 m double monochromator^18^. This justifies the different observations of two types of measurements.

### Super-hydrogenic Dopant

**Figure 3:**
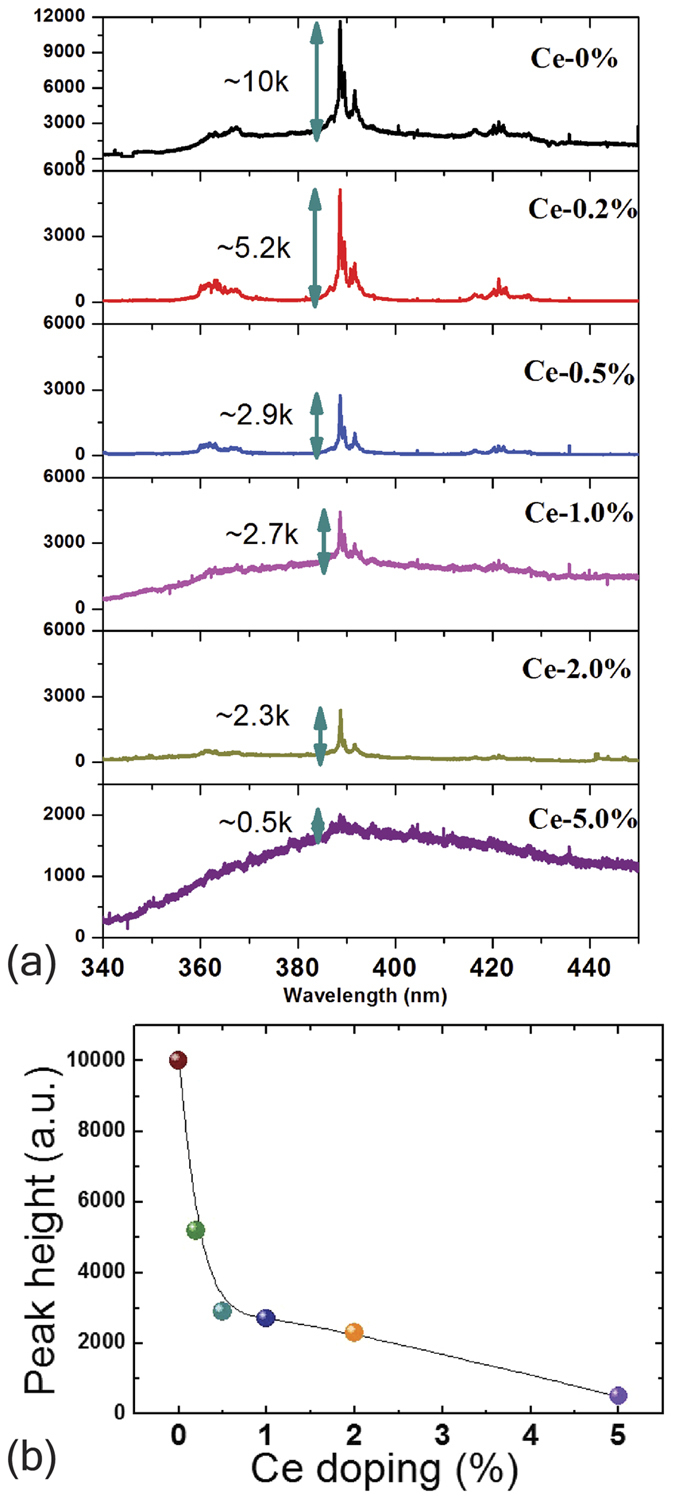
Room Temperature PL quench. (**a**) the systematic PL intensity quench of 390 nm PL emission line in polycrystalline bulk NGO with Ce doping (**b**) 390 nm PL intensity peak plotted against Ce doping percentage.

Figure 3 discusses the systematic drop in PL intensity leading to a quench of the PL lines. Figure 3(b) shows the peak height of the 390 nm PL line as a function of Ce doping in NGO poly-crystalline bulk samples. Here we have focused at the most prominent PL peak of 390 nm which most likely originates from ^4^D_3/2_ → ^4^I_11/2_ and/or ^2^P_3/2_ → ^4^I_9/2_ transition of Nd^3+^. We find that initially the PL peak height of the 390 nm line in pure NGO poly-crystalline sample was ≈10 k counts, which drastically drops to almost half i.e. ≈5.2 k at only 0.2% elemental doping of Ce^4+^ and finally the 390 nm PL peak intensity completely suppressed by only 5% elemental doping of Ce^4+^. Figure 3(b) shows a clear near-exponential systematic decrease of the peak intensity as the Ce elemental doping level is increased. As Ce^4+^ replaces Nd^3+^, it donates an extra electron to the system. As this electron doping forms a deep level it does not contribute to the electrical conductivity of the material. Using the doping-dependent drop in the peak height shown in Fig. 3(b) we can calculate the effective Bohr radius of the extra dopant electron.

Using Avogadro number, molecular weight and density of NGO we can calculate the number of NGO molecules per unit volume that comes out to be ~3.46 × 10^22^/cm^3^. We find that the total PL almost quenches near 5% Ce doping – so that means effective Bohr radii of 5% Ce atoms envelops almost all the Nd^3+^ ion centers which are clearly the source of PL in this study. This assumes that all Ce ions are in the 4+ state. To determine the valence of the Ce we refer to Fig. 1(d), where we have calculated the area under the curve for all these XPS peaks and find that 45% of the Ce is in the 4+ state while 55% is in 3+. This means that only 45% of Ce doping in the 5% Ce-NGO sample contributes to PL quench, corresponding to an “effective” doping of approximately 2.2% and an electron concentration of ~3.81 × 10^20^/cm^3^ related to the quenching, so the volume occupied by each Ce dopant electron comes out to be ~2.62 × 10^−21^ cm^3^. This value further leads to an effective Ce^4+^ electronic radius of ~8.5 Å if we assume all PL quench happened precisely at 5% Ce doping level.

The simple calculation procedure described above can be represented by the mathematical equation shown below:


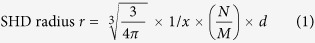


where

*x* – Dopant concentration required to completely quench the PL signal.

N – The Avogadro number;

M – The molecular weight of the material (here it is NdGaO_3_);

*d* – The density of the material.

The mathematical relationship clearly shows that the SHD radius varies inversely with the cube root of *x* if the material system is unchanged.

Physically we can also visualize the phenomena – as we know all the Nd^3+^ ion centres acts as the source the PL and effective radius of the Ce^4+^ dopant electron is acting like a cloak covering some n number of Nd^3+^ ion centers hence reducing the intensity of the PL peak. If lower dopant concentration causes complete PL quench then it clearly means effective SHD Ce^4+^ radius has to be larger.

Now, if we assume that the precise doping level (where all the PL quenches) lies somewhere between 2 to 5% of Ce^4+^ doping then we find that the SHD radius spans from 0.85 nm (for 5% doping) to 1.15 nm (for 2% doping). Using the free space Rydberg radius value of 0.5 Å and considering the bulk dielectric constant of NGO of ~20.2 we estimate a SHD radius of 1.01 nm which lies exactly in the middle of our experimental range of 0.85–1.15 nm.

## Discussion

In this study we have reported the PL properties of NGO single crystals and polycrystalline bulk samples where UV (~360 and ~390 nm) and blue emission (~420 nm) lines were observed. The observed UV and blue emission can be understood based on the energy level diagram of the Nd^3+^ ion. Following the observation of NGO PL emission we have seen a systematic quench of one sharp UV line (390 nm) with very small amount of Ce^4+^ doping in the place of Nd^3+^. This PL quench has been explained by the novel idea of a super-hydrogenic dopant where the SHD radius is enhanced by the large dielectric constant of the host. The experimentally derived range of SHD radius comes out to be 0.85 nm to 1.15 nm where the predicted radius of 1.01 nm (from the bulk dielectric constant of the host) in the middle of that range. Thus using PL quenching we may able to estimate the Bohr radius of other super hydrogenic dopants in wide band-gap solids with large dielectric constants with the possibility of new functionalities.

In final remark, we would like to add that although SHD means super-hydrogenic dopant – it clearly has nothing to do with doping using hydrogen atom/ions. Any element which can act as an electronic dopant can be a SHD in a host with large dielectric constant. However, in terms of the host material we need rare-earth based oxides and perovskite materials like NdGaO_3_, NdAlO_3_ or Lu_2_O_3_, Nd_2_O_3_, Gd_2_O_3_ etc. to study SHD using the sharp photo luminesces from the rare earth atoms. This sharp atomic PL lines and their subsequent quench (with doping) are very crucial to study, understand and quantify the effect of SHD and its dimension. Other perovskite systems (widely used in oxide-electronics research) like SrTiO_3_, KTaO_3_ etc. may not be the ideal systems to study SHD phenomena as they usually have broad PL emission originating from band-edge or defect states.

## Methods

### Sample Preparation

In this study we have initially used (1 1 0) oriented NGO single crystal (5 × 5 × 0.5 mm^3^) from CrysTec GmbH, Germany, for the single crystal study of the NGO pure system. Further, we have prepared one pure NGO poly-crystalline pellet and five different Ce doped NGO poly-crystalline pellets. For the pure NGO bulk sample we have used the high purity (99.995% or higher) powders of Nd_2_O_3_ and Ga_2_O_3_. Moreover, for other five doped NGO bulk samples high purity CeO_2_ powder was used along with Nd_2_O_3_ and Ga_2_O_3_ powders. At first, the powders are mixed for about 1 h by hand using mortar and pestle and the mixed powder was calcined at 1000 °C for ≈12 h in an oven at a closed environment in atmospheric pressure. The calcined powder is then further ground in mortar and pestle again and then subsequently pressed into 1-in diameter pellets by hydraulic press. Finally, the pellets were sintered at 1350 °C under the same atmospheric pressure for ≈16 h.

### Measurement and characterization

PL properties of NGO single crystals and thin films were studied by exciting with the 325 nm line of a He-Cd laser with a spot size of ~800 nm and a power of 10 mW, (laser intensity ~1.5 MW/cm^2^) and recorded using a JY-Horiba LabRAM HR spectrometer. Further elemental composition, valence and structural analyses were done using Rutherford backscattering spectroscopy ((using 3.5 Mev singletron accelerator)), secondary ion mass spectroscopy (using ToF-SIMS-IV from ION-TOF GmbH), x-ray photoelectron spectroscopy (using Axis Ultra Delay Line Detector from Kratos Analytical) and x-ray diffraction (using Empyrean powder XRD system from PANalytical).

## Additional Information

**How to cite this article**: Ghosh, S. *et al.* Origin and Quenching of Novel ultraviolet and blue emission in NdGaO3: Concept of Super-Hydrogenic Dopants. *Sci. Rep.* **6**, 36352; doi: 10.1038/srep36352 (2016).

**Publisher’s note**: Springer Nature remains neutral with regard to jurisdictional claims in published maps and institutional affiliations.

## Supplementary Material

Supplementary Information
