# Supplementary material for: Origin and Quenching of Novel ultraviolet and blue emission in NdGaO3: Concept of Super-Hydrogenic Dopants
Source: Sci Rep. 2016 Nov 3;6:36352. doi: 10.1038/srep36352 (PMC5093437; doi:10.1038/srep36352)
Supplement: Supplementary Information [file srep36352-s1.pdf]

Origin and Quenching of Novel ultraviolet and blue emission in NdGaO3: Concept of Super-Hydrogenic Dopants

S. Ghosh[1#*], S. Saha[1,2#⊥], Z. Q. Liu[1,2,7*], M. Motapothula[1], A. Patra[1], N. Yakovlev[5], Y. Cai[6], S. Prakash[1], X. H. Huang[1,3,5], C. B. Tay[1], C. X. Cong[4], T. Bhatt[5], S. B. Dolmanan[5], J. Q. Chen[1,3], W. M. Lü[1], Z. Huang[1], S. Tripathy[5], S. J. Chua[3], T. Yu[4], M. Asta[6], A. Ariando[1,2,*] T. Venkatesan[1,2,3],

[1]*NUSNNI-Nanocore, National University of Singapore, 117411 Singapore*

[2]*Department of Physics, National University of Singapore, 117542 Singapore*

[3]*Department of Electrical and Computer Engineering, National University of Singapore, 117576 Singapore*

[4]*Divsion of Physics and Applied Physics, School of Physical and Mathematical Sciences, Nanyang Technological University, 637371 Singapore*

[5]*Institute of Materials Research and Engineering, A*STAR (Agency for Science, Technology, and Research), 117602 Singapore*

[6]*Department of Materials Science and Engineering, UC Berkeley, 210 Hearst Ave, Berkeley, CA 94720, United States*

[7]*School of Materials Science and Engineering, Beihang University, Beijing 100191, China*

[#]These authors contribute equally to this work.

*To whom correspondence should be addressed.
E-mail: nnisg@nus.edu.sg, zhiqi@buaa.edu.cn, ariando@nus.edu.sg;

⊥ Presently at Indian Institute of Science Education and Research, Bhopal, Madhya Pradesh, 462066, India

SUPPORTING INFORMATION

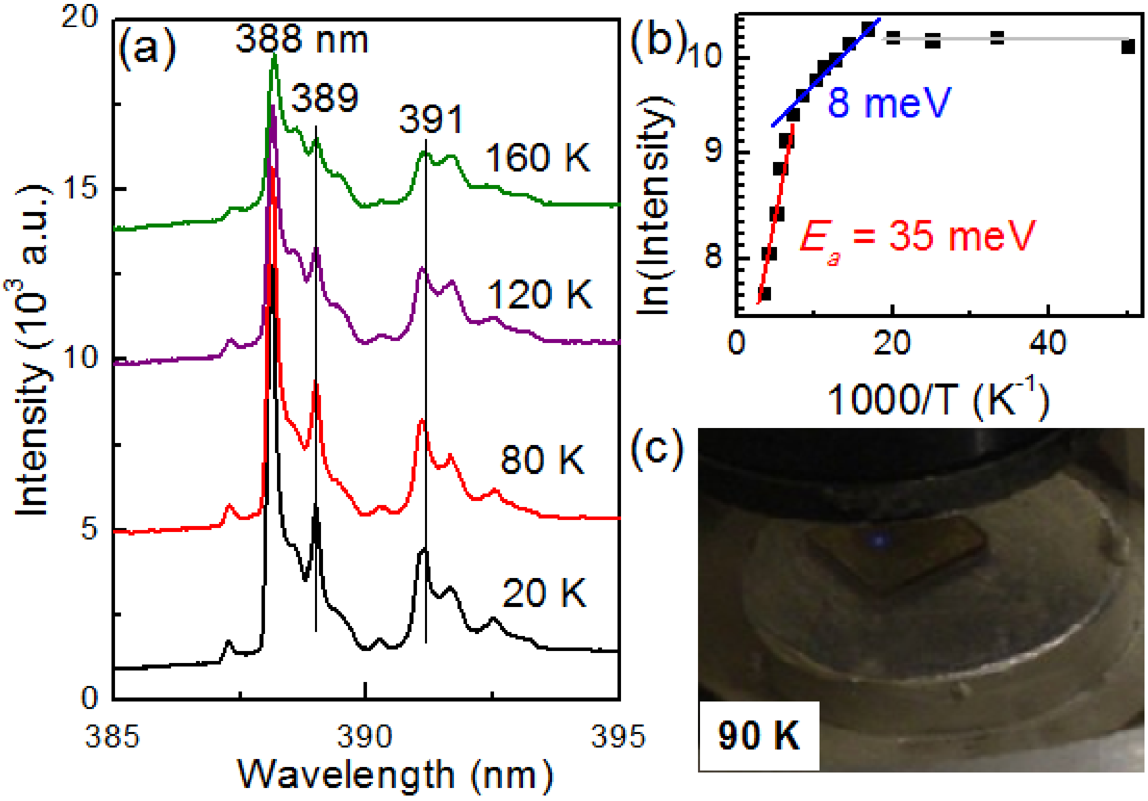

Figure S1: Low-temperature PL properties of a NGO single crystal. (a) Low-temperature PL spectra of a NGO single crystal in the range of 380 to 396 nm. (b) Arrhenius plot of the integrated intensity of the 388 nm emission peak. Solid lines are guides to the eyes. (c) Blue emission of NGO at 90 K. The sample was excited by a 325 nm laser with the laser spot size of 800 nm and the intensity of 1 MW/cm^2. As the temperature was lowered, emission peaks did not shift but the emission intensity of all peaks was pronouncedly enhanced. The intensity of the 388 nm peak at 20 K is increased by a factor of ten compared with that at room temperature. In addition, the emission peaks at 389 and 391 nm can also be seen in the low-temperature spectra. As the temperature increases, the recombination rate and accordingly the emission intensity decrease due to the thermal activation of photo-excited electrons. Such a process can be described by

$$I(T) = I_0/[1 + \exp(-E_a/kT)]$$

where E_a is the activation energy. The integrated intensity can be well fitted by the above formula above 140 K and the fitted activation energy is 35 meV.

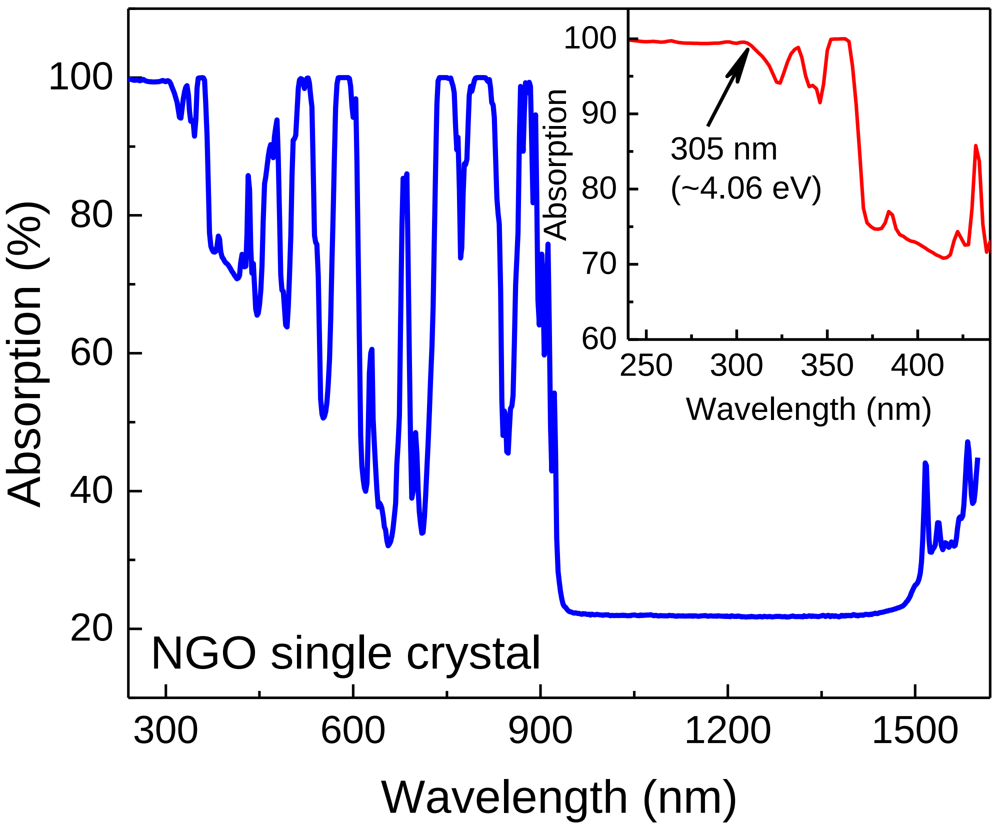

Figure S2: Room temperature absorption Spectra of NGO crystals. Absorption spectrum of a NGO single crystal in the wavelength range of 300 to 1500 nm range. In the inset we shown the zoom-in spectrum below 440 nm.

The band gap of NGO is ~4.06 eV found from the UV-VIS analysis, which is larger than the excitation energy of the 325 nm laser (3.8 eV). Therefore, the PL emission observed in NGO single crystals is obviously not band gap emission. Instead, the emission peaks observed in NGO can be readily understood by looking into the energy levels of Nd^{3+} shown in Fig. 2(b) of the main text.

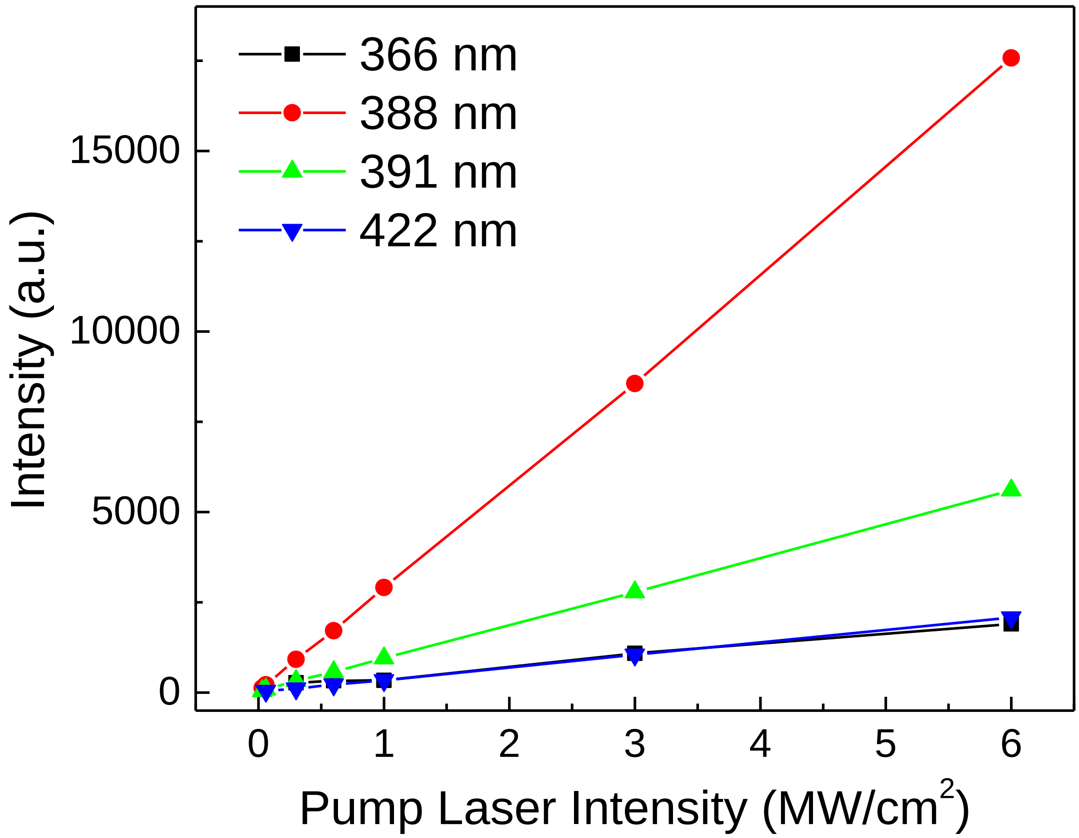

Figure S3: Room-temperature PL emission of NGO single crystals. Room-temperature emission intensity of different peaks as a function of the pump laser intensity. The intensity of all the emission peaks show ideally linear dependence on the pump intensity (partially shown in the Figure above). This reveals that the emission in NGO is potential for laser energy calibrator.

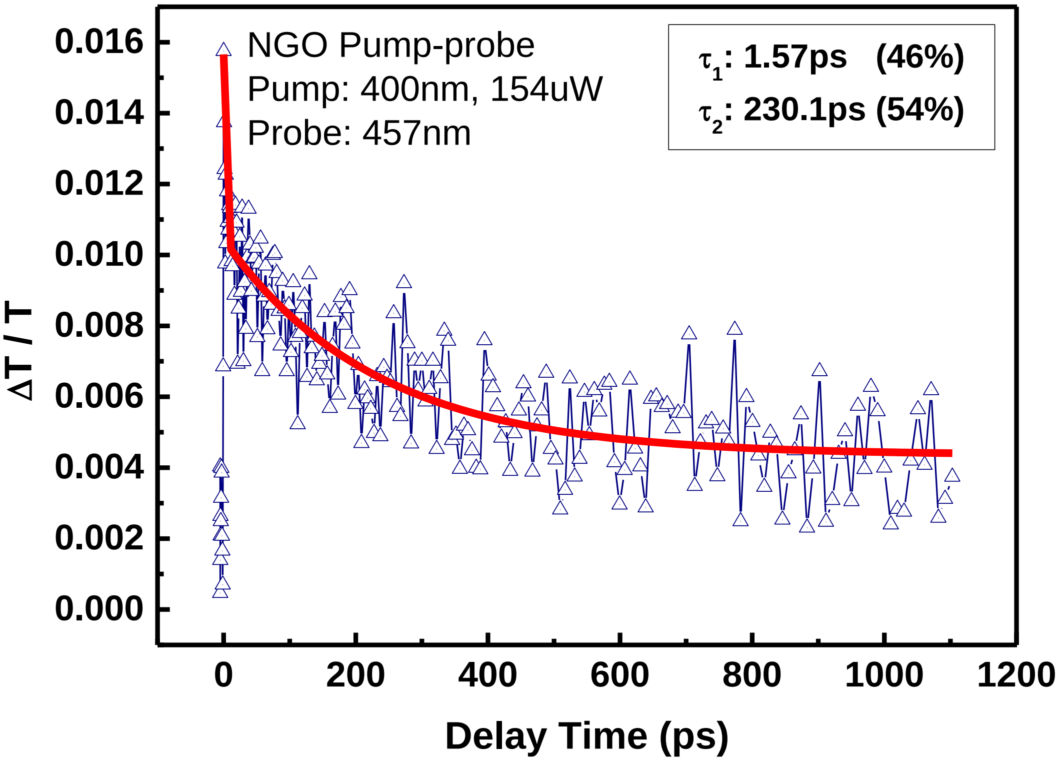

Figure S4: Room-temperature Pump-probe study of NGO single crystal.

Room-temperature pump-probe spectroscopy was carried using pump wavelength of 400 nm (with 145 μW energy) and probe wavelength of 457 nm (with 10 μW energy). Both the pump and probe pulse have the pulse duration of 100 fs and with the focus spot size diameter of 350 and 100 um, respectively. The study reveals two different excitations with different range of decay time – one in the range of 1.57ps and another two order of magnitude higher, in the range of 230 ps. The fast and slow decay shown almost the same percentage (46% and 54%), which is consistent with the PL study, where the 420 and 422 nm have almost the same intensity at room temperature [Fig. 2(a)]. The fast decay time may indicate that the electron transition between the higher state ($^4D_{3/2}$) to adjacent state ($^2P_{3/2}$) with lifetime of about 1.57 ps. The slow decay time indicate the PL emission lifetime, where the electron transition from higher state $^4D_{3/2}$ to lower state $^4I_{13/2}$ with lifetime of about 230 ps [Fig. 2(b)].
